# Nucleation and stability of skyrmions in three-dimensional chiral nanostructures

**DOI:** 10.1038/s41598-020-78838-6

**Published:** 2020-12-10

**Authors:** Yan Liu, Na Cai, Xingxing Yu, Shengjie Xuan

**Affiliations:** College of Sciences, Northeastern University, Shenyang, 110819 People’s Republic of China

**Keywords:** Magnetic properties and materials, Spintronics

## Abstract

We studied the magnetization evolution in three-dimensional chiral nanostructures, including nanotubes and circularly curved thin films, by micromagnetic simulations. We found that in a nanotube skyrmions can be formed by broken of the helical stripes on the left and right sides of the nanotube, and the formation of skyrmions doesn’t correspond to any abrupt change of topological number. Skyrmions can exist in a large range of magnetic field, and the thinner nanotube has a larger field range for skyrmion existence. The configuration of a skyrmion in nanotubes is different from the one in thin film. From the outer to the inner circular layer, the size of the skyrmion becomes larger, and the deformation becomes more obvious. In circularly curved magnetic films with fixed arc length, there are three kinds of hysteresis processes are found. For the curved films with a large radius, the magnetization evolution behavior is similar to the case in two-dimensional thin films. For the curved films with a small radius, the skyrmions are created by broken of the helical stripes on the left and right sides of the curved film. For the curved film with a medium radius, no skyrmion is formed in the hysteresis process.

## Introduction

The topic of magnetism in curved geometry has attracted much attention in modern magnetism research. Curvature effect has been emerged as an efficient means to impact the statics and dynamics of magnetic texture^1–3^. It may bring the emergence of unconventional spin textures, where novel physical effects comprising geometry, topology, and chirality^3–7^. It has demonstrated that curvature will induce two additional energies: geometrically induced-anisotropy energy and effective Dzyaloshinskii-Moriya interaction (DMI) energy^3,4,8^. These additional energies excite many striking novel properties in three-dimensional curved magnetic thin films, wires, and nanotubes, such as magnetochiral effects^9,10^, topologically induced magnetic patterns^11^, and Spin-Cherenkov effect of spin waves^12^.

Magnetic skyrmions are stable spin textures with the magnetic moments forming a twist structure. The key characteristic is they are topological protected. They have been widely considered as data carriers in spintronics^13–18^. It is known that skyrmions are rather peculiar topological magnetic structures and they have been observed in several classes of magnetic materials without inversion symmetry^13–26^. Both DMI energy and anisotropy energy have an obvious effect on the stability of skyrmions^14,27^. Therefore, the interplay between skyrmions and curvature is worthy to investigate. It was reported that skyrmions may be stabilized by curvature effects only in a spherical shell^28,29^, and skyrmions can be the ground state when its radius is comparable with the size of curvilinear defect of a thin magnetic film^30^. More recently, Wang et al. reported that skyrmions can move in magnetic nanotubes in presence of very large current density without annihilation^31^, and we also demonstrated that skyrmions can exist in a nanotube^32^.

In this work, we introduce curvature into chiral magnetic materials to investigate the magnetization evolution in three-dimensional chiral nanostructures, including nanotubes and curved thin films. We use micromagnetic simulation to describe how the characteristic properties such as the skyrmion nucleation and annihilation and its structure depending on the size of nanostructures. Indeed, those three-dimensional magnetic structures with complicated geometries and different sizes have been fabricated by various chemical and physical approaches^7,33–35^, which may make our simulations be experimentally measured.

## Method

The magnetization dynamics in chiral nanostructures is described by the Landau–Lifshitz–Gilbert (LLG) equation:

1 $$ {\dot{\mathbf{m}}} = - \gamma {\mathbf{m}} \times {\mathbf{H}}_{{{\text{eff}}}} + \alpha {\mathbf{m}} \times {\dot{\mathbf{m}}}, $$

where $\gamma$ is the gyromagnetic ratio, $\alpha$ is the Gilbert damping constant, and ${\mathbf{m}}$ is the unit vector representing the orientation of the magnetization. The effective magnetic field is given by ${\mathbf{H}}_{{{\text{eff}}}} = - {1 \mathord{\left/ {\vphantom {1 {(\mu_{0} M_{s} )}}} \right. \kern-\nulldelimiterspace} {(\mu_{0} M_{s} )}}({{\partial W} \mathord{\left/ {\vphantom {{\partial W} {\partial {\mathbf{m}}}}} \right. \kern-\nulldelimiterspace} {\partial {\mathbf{m}}}})$, where *W* is the magnetic energy of the nanodisk consisting of exchange, DMI, magnetostatic, and Zeeman energy terms. The LLG equation is solved by using the Mumax code^36^. In the simulation, the nanotube is discretized into many small cells with a cell size of $2 \times 2 \times 2{\text{ nm}}^{3}$. The material parameters used for the simulations corresponding to FeGe^37^: saturation magnetization $M_{s} = 3.84 \times 10^{5} \;{\text{A/m}}$, the exchange constant $A = 8.78 \times 10^{ - 12} \;{\text{J/m}}$, DMI constant $D = 1.58 \times 10^{ - 3} \;{\text{J/m}}^{2}$,and the Gilbert damping constant $\alpha = 0.2$.

In order to clarify different observed magnetization configurations in three-dimensional nanostructures, we calculated the topological number of the system. As is well known, in a two-dimensional plane the topological number is defined as

2 $$ Q = \frac{1}{4\pi }\int {{\mathbf{m}} \cdot \left( {\frac{{\partial {\mathbf{m}}}}{\partial x} \times \frac{{\partial {\mathbf{m}}}}{\partial y}} \right)} dxdy. $$

**Figure 1:**
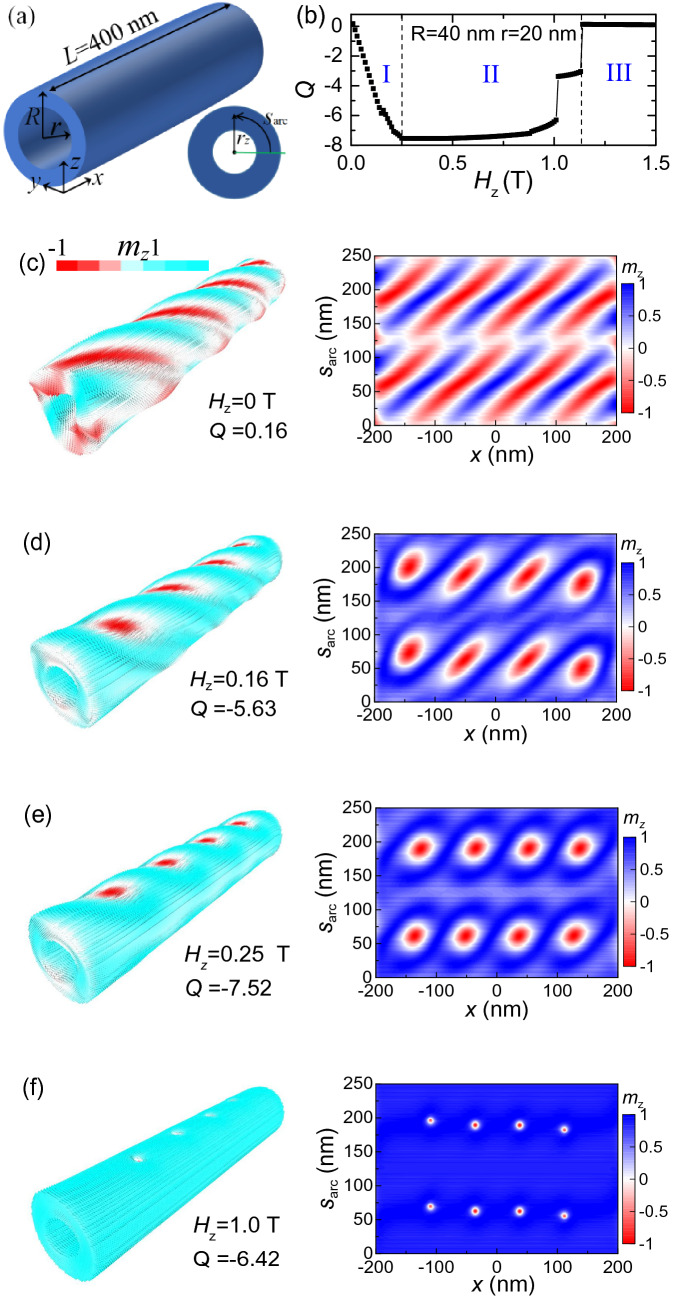
(**a**) The sketch of the nanotube. Its length is fixed at *L* = 400 nm, its outer and inner radius are denoted by *R* and *r*, respectively. The Cartesian coordinates and the cylindrical coordinates used for the analytic study are sketched in the inset of this figure, where *r*_*z*_ is the radius of a circular layer, and $s_{arc}$ is the arc length from the line *y* = 0 for a fixed *r*_*z*_. (**b**) The topological number as a function of *H*_*z*_ in a nanotube when a *z*-direction magnetic field is applied, where *R* = 40 nm and *r* = 20 nm. Three regions are depicted in this figure, where I is the region of twisted helical stripe state, II is the region of skyrmions, and III is the region of ferromagnetic state. (**c**)–(**f**) The snapshot of the magnetic state at different *H*_*z*_. The left panel is the three-dimensional magnetic configurations of the nanotube. The arrows represent the magnetic moment, and the color encodes the *z* component of the magnetization, The color scale is shown on the top. The right panel is *m*_*z*_-image in *x*-$s_{arc}$ plane for *r*_*z*_ = 39 nm.

The topological number is defined as a measure of the wrapping of **m** around a unit sphere, which allows to show the change of the magnetization configuration in the system, for example, the formation of skyrmions^38^. Also, it can be used to counting the number of skyrmions. In our model, the magnetic states reside in a curved film. To calculate the topological number in such geometry, we first choose the magnetization data in a circular layer with a fixed radius *r*_z_ (Fig. 1a), and then we calculate the topological number of the skyrmion in this circular layer as

3 $$ Q = \frac{1}{4\pi }\int {{\mathbf{m}} \cdot \left( {\frac{{\partial {\mathbf{m}}}}{\partial x} \times \frac{{\partial {\mathbf{m}}}}{\partial s}} \right)} dxds, $$

where $ds = \sqrt {\left( {dy} \right)^{2} + \left( {dz} \right)^{2} }$ is the differential arc.

In this paper, the sample is a three-dimensional geometry. To show the magnetic states in this geometry more clearly, besides the three-dimensional images, we also show the *m*_*z*_-image in a two-dimensional plane. We first choose the data in a circular film that satisfy the condition $r_{z} - 2\,{\text{nm}} < \sqrt {y^{2} + z^{2} } < r_{z}$. Then we take *x* as X-axis, $s_{arc}$ as Y-axis, where $s_{arc}$ is the arc length from the line *y* = 0, as shown in the inset in Fig. 1c. At last, we plot the *m*_*z*_-image in the *x*-$s_{arc}$ plane.

## Results and discussion

### Nanotube

Figure 1a shows the geometry structure of a nanotube. Its length is fixed at *L* = 400 nm. The outer radius and the inner radius are expressed by $R$ and $r$, respectively. The thickness of the nanotube equals to *R*-*r*.

To begin, we consider the magnetization evolutions in a nanotube under the effect of magnetic field. Figure 1b shows the typical field-driven evolution of topological number *Q* in a nanotube, where *R* = 40 nm and *r* = 20 nm. Discontinuous changes in the *Q* curve indicate that the system presents different spin textures at different magnetic fields. Three different states are evolved with increasing of the magnetic field. Figure 1c–f shows the magnetic states at different *H*_*z*_. At *H*_*z*_ = 0, it is a twisted helical stripe state, as the left panel in Fig. 1c shown. The corresponding *m*_*z*_-image of the nanotube in *x*-$s_{arc}$ plane is shown in the right panel of Fig. 1c. It shows the typical helix state, which is similar with the state in thin films^19^. For this state, its topological number *Q* = 0.16, which is close to 0. With the increasing of *H*_*z*_, the topological number decreases continuously. Meanwhile, the magnetizations on the left and right sides of the nanotube rotate to the *z*-direction, which narrows down the helical stripes at these positions, and the magnetizations whose orientation ia along the –*z*-axis (red regions) are distributed mainly on the top and bottom of the nanotube (Fig. 1d). At *H*_*z*_ = 0.25 T, the helical stripes are completely broken on the left and right sides of the nanotube (Fig. 1e), the value of *Q* decreases to − 7.52, and after that *Q* almost doesn’t change with *H*_*z*_. From the right panel in Fig. 1e, we can see that eight skyrmions are formed, and they are evenly distributed on the top and bottom of the nanotube. The eight skyrmions can exist until *H*_*z*_ = 1.0 T, and the size of the skyrmions decreases obviously with increasing of the *H*_*z*_ (Fig. 1f). The topological number *Q* jumps to about 4 when *H*_*z*_ = 1.01 T, which corresponds to the annihilation of four skyrmions that near the boundary, and the other four skyrmions annihilate at *H*_*z*_ = 1.14 T. The system changes to the ferromagnetic state.

It is worthy to note that in the nucleation process the topological number *Q* gradually increases from 0 to about 8 without any abrupt jump. This is different from the case in thin films. In thin films, the nucleation of skyrmions usually corresponds to a sudden jump of *Q*^39^. The skyrmions in nanotubes are created by broken of the helical stripes on the left and right sides of the nantotube, so the periodicity of the helical stripes determines the skyrmion number. According our simulation, we find the periodicity of the helical stripes is related to thickness and the radius of the nanotube. For the nanotube with a smaller outer radius and a thinner thickness, it has the highest density of the helical stripes, which corresponding to more skyrmions. Instead, for the nanotube with a larger outer radius and a thicker thickness, it has the lowest density of the helical stripes and the least skyrmion number.

**Figure 2:**
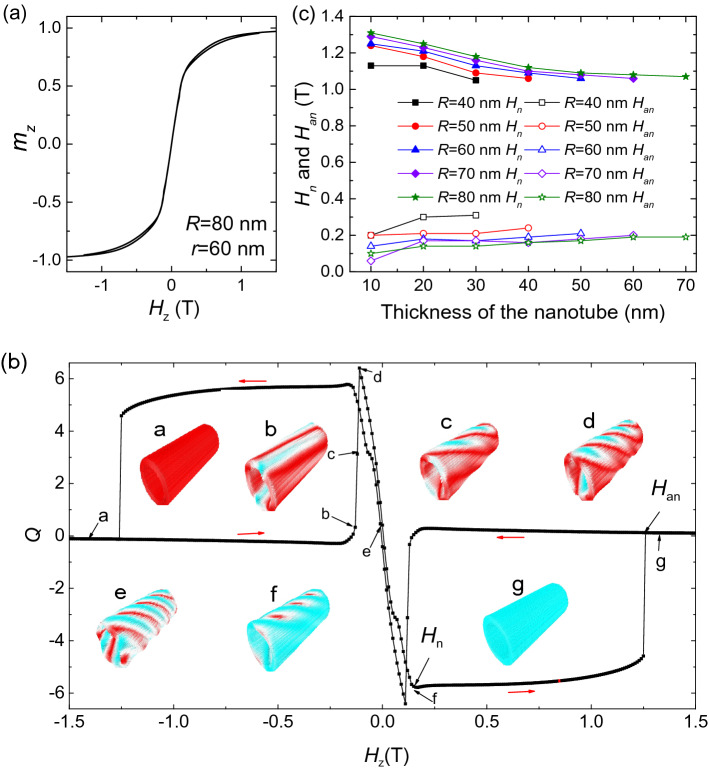
(**a**) The typical hysteresis loop of a nanotube (*R* = 80 nm, *r* = 60 nm) under a *z*-direction magnetic field. (**b**) The topological number as a function of *H*_*z*_ and the evolution of the magnetic structure during this process. (**c**) The size dependence of nucleation field *H*_n_ and the annihilation field *H*_an_ for nanotubes with different radii.

Then we show the hysteresis behaviors. Figure 2a shows the typical hysteresis loop of a nanotube, where *R* = 80 nm and *r* = 60 nm. The corresponding *Q*–curve and the typical magnetic states are shown in Fig. 2b. Starting from the uniformly magnetized system at *H*_*z*_ = − 1.5 T (a). At *H*_*z*_ = − 0.21 T, small stripes are formed in the nanotube. When *H*_*z*_ increases from − 0.21 to − 0.19 T, more stripes are formed, which increases the value of *Q* quickly to 6.2 (b–d). After that, the small stripes gradually evolve to twisted helical stripes, this evolution decreases the topological number from 6.2 to 0 (d–e). Then, the twist helical stripes become narrow on the left and right side of the nanotube. At *H*_*z*_ = 0.16 T, they are completely broken on the left and right sides of the nanotube, skyrmions are formed in the nanotube (f). We define the magnetic field that the skyrmions are formed as the nucleation field *H*_n_. The skyrmions can stay a wide field range between *H*_*z*_ = 0.15 T and 1.25 T. At last, at *H*_*z*_ = 1.26 T, the skyrmions annihilate, the configuration of the nanotube changes to a ferromagnetic state (g). Here we define the magnetic field that the skyrmions annihilate as the annihilation field *H*_*an*_.

Figure 2c shows the dependence of the skyrmion nucleation and annihilation field on size. The nucleation field decreases with decreasing of the nanotube thickness, however, the annihilation field increases with decreasing of the nanotube thickness. This makes the thinner nanotube has a larger field range for the existence of skyrmions. On the other hand, the nanotube has a larger radius may have a smaller nucleation field and a larger annihilation field. For example, for the nanotube (*R* = 40 nm) with thickness of 30 nm, *H*_n_ = 0.31 T and *H*_an_ = 1.05 T. However, for the nanotube (*R* = 80 nm) with thickness of 10 nm, *H*_n_ decreases to 0.1 T, and *H*_an_ increases to 1.31 T.

**Figure 3:**
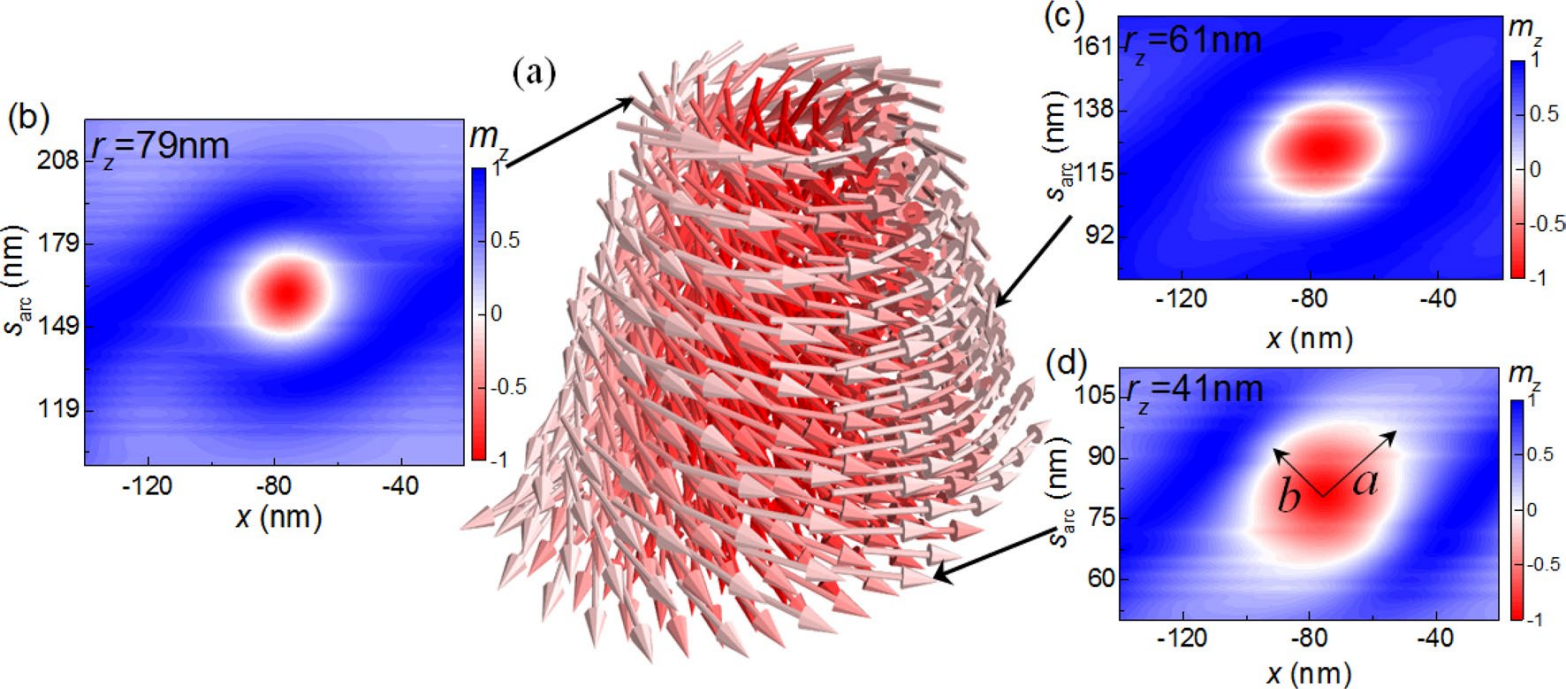
(**a**) Vector plot of the skymion state in a nanotube (*R* = 80 nm and *r* = 40 nm) at *H*_*z*_ = 0.37 T. (**b**)–(**d**) *m*_*z*_-image in *x*-$s_{arc}$ plane for different *r*_*z*_.

Now let us consider the skyrmion configuration in a nanotube. In thin films, the shape of skyrmion is circular, its magnetizations are isotropic distributed in different directions^40^. However, in a nanotube the configuration of skyrmion is different. Figure 3a shows the vector plot of the skyrmion state in a nanotube (*R* = 80 nm and *r* = 40 nm) at *H*_*z*_ = 0.37 T. We can find that the shape of the skyrmion is a circular truncated cone. Figure 3b–d shows the *m*_*z*_-image in *x*-$s_{arc}$ plane with different *r*_*z*_. It is shown that that shape of the skyrmion in each circular layer is ellipse. From the inner circular layer to the outer circular layer, the size changes a lot. In order to better describe the size of skyrmion we use ellipse to fit the boundary of the skyrmion (the isoline with *m*_*z*_ = 0). The semimajor and semiminor axis of the ellipse are represented by *a* and *b*, respectively. For $r_{z} = 79{\text{ nm}}$, *i.e.* the outer circular layer in the nanotube, $a = 14.7\,{\text{nm}}$ and $b = 13.7\,{\text{nm}}$. When *r*_*z*_ decreases to 61 nm, i.e. the middle circular layer, $a = 17.4\,{\text{nm}}$ and $b = 16.1\,{\text{nm}}$. Further, for $r_{z} = 41\,{\text{nm}}$, i.e. the inner circular layer in the nanotube, $a = 24.8\,{\text{nm}}$ and $b = 19.9\,{\text{nm}}$. These data indicate that from the outer circular layer to the inner circular layer, the skyrmion becomes larger, and the deformation becomes more obvious.

**Figure 4:**
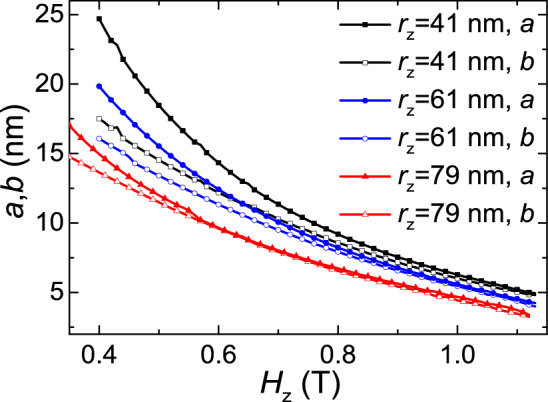
Variation of the skyrmion size with *H*_*z*_ for different *r*_*z*_ in the nanotube with *R* = 80 nm and *r* = 40 nm.

Figure 4 shows the dependence of the skyrmion size on the applied magnetic field $H_{z}$ in circular films with different *r*_*z*_. We can see that the difference between $a$ and $b$ are large in low field, but when the magnetic field increases, *a* and *b* become almost equal. Also, the size of the skyrmion is large and the deformation is obvious in the inner circular layers. For instance, for $r_{z} = 41\,{\text{nm}}$, at $H_{x} = 0.4\,{\text{T}}$, $a = 24.8\,{\text{nm}}$_,_ $b = 19.9\,{\text{nm}}$, and the value of $a - b = 4.9\,{\text{nm}}$. However, for $r_{z} = 79\,{\text{nm}}$, *a*, *b* and $a - b$ decrease to 7.5 nm, 7.3 nm and 0.2 nm at $H_{x} = 0.9\,{\text{T}}$.

### Curved thin film

**Figure 5:**
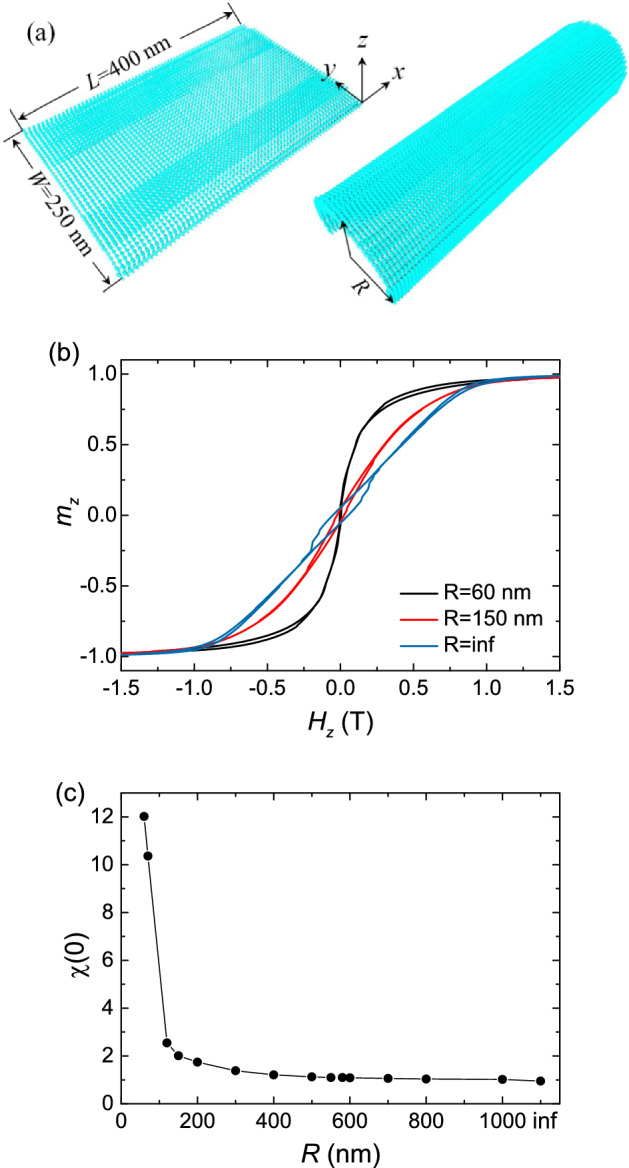
(**a**) The sketch of the curved film. The size of the film is fixed at length of 400 nm, width of 250 nm, and thickness of 10 nm. Then, it is curved to a circularly curved film with a fixed radius *R*, where *R* varies from 40 nm to infinity. (**b**) The hysteresis loops for the curved films with *R* = 60, 150 and inf, respectively. (**c**) The susceptibility at *H*_*z*_ = 0 as a function of *R*.

We also study the magnetization evolution in curved magnetic films. The model is shown in Fig. 5a. We first choose a thin film (the left panel). Its length *L* = 400 nm, width *W* = 250 nm, and thickness *H* = 10 nm. Then, we curve the thin film to a circularly curved film with a fixed radius *R*, where *R* varies from 40 nm to infinity. We calculate the hysteresis behaviors under a magnetic field along *z*-axis for these circularly curved films.

Figure 5b shows the typical hysteresis loops for the curved films with *R*. We find that with decreasing of *R*, the remanence increases and the coercivity decreases. The susceptibility at *H*_*z*_ = 0 ($\chi (0)$) increases with decreasing of *R*. Figure 5c shows the value of $\chi (0)$ as a function of *R*. We can find that when R > 150 nm, $\chi (0)$ slightly increases with decreasing of *R*. However, when R < 150 nm, $\chi (0)$ rapidly increases with decreasing of *R*. Particularly, as *R* decreases to 70 nm, $\chi (0)$ increased to 10.4. For the case of the two-dimensional thin film (*R* = inf), the hysteresis loop that we calculated is similar to the measurement results in Ref.^41^.

**Figure 6:**
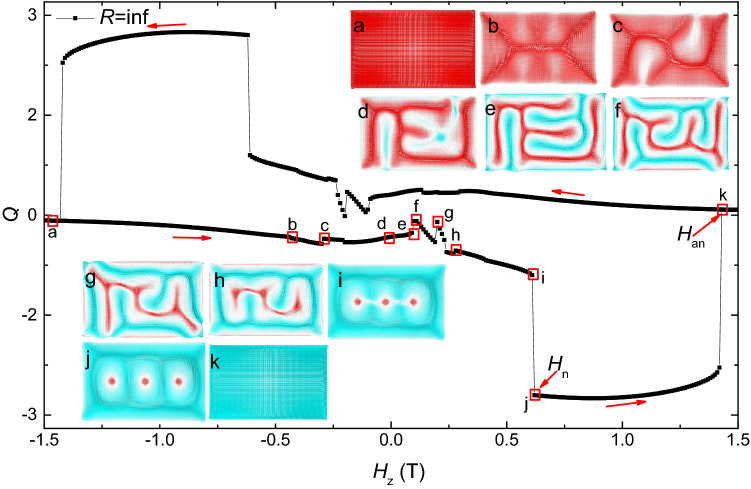
The topological number as a function of *H*_*z*_ and the evolution of the magnetic structure during this hysteresis process in the thin film (*R* = inf).

**Figure 7:**
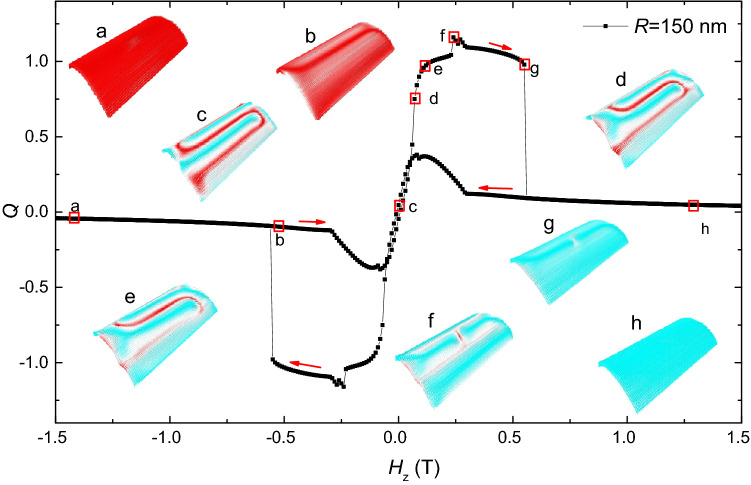
The topological number as a function of *H*_*z*_ and the evolution of the magnetic structure during this process in the circular curved film with *R* = 150 nm.

**Figure 8:**
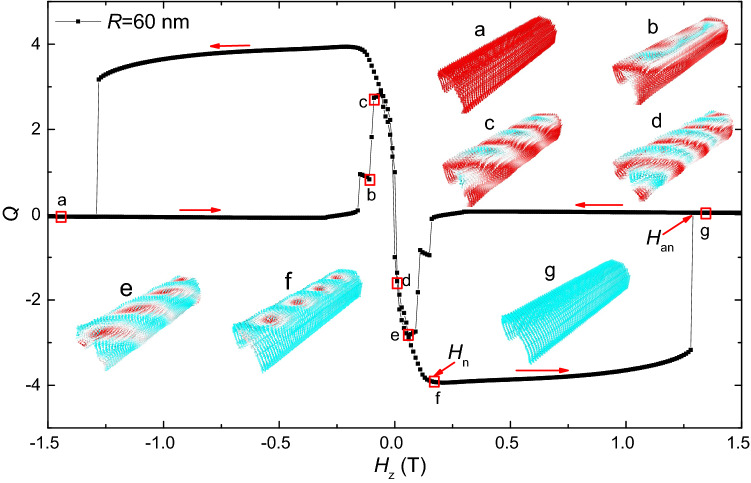
The topological number as a function of *H*_*z*_ and the evolution of the magnetic structure during this process in the circular curved film with *R* = 60 nm.

Next, we will show the evolution of magnetic states during these hysteresis processes. There are three different types of evolution process. Figures 6, 7 and 8 show the typical *Q*-curves for these three types, and the represented magnetic states are shown as insets.

For $R > 600\,{\text{nm}}$, the evolution of magnetic states is similar to the case of thin film ($R = \inf$). A typical field-driven evolution of the magnetic state is shown in Fig. 6, where $R = \inf$, i.e. the rectangular thin film. When magnetic field increases from − 1.5 to 0.11 T, the magnetic state gradually changes from the uniformly magnetized state to the maze domains (a–e). The topological number changes slightly, it almost equals to 0. Then when the magnetic field gradually increases from 0.11 to 0.61 T, the topological number shows some small jumps and slowly decreases, the maze domain gradually shrinks to a distorted nanostripe (f–i). At *H*_*z*_ = 0.62 T, the topological number shows a sudden jump, meanwhile the distorted nanostripe breaks, three skyrmions are formed in the thin film (i, j). The skyrmions can exist in a wide field range between *H*_*z*_ = 0.62 T and 1.41 T. At last, at *H*_*z*_ = 1.42 T, the skyrmions annihilate, the configuration of the thin film changes to the ferromagnetic state (k).

For $150\,{\text{nm}} \le R < 600\,{\text{nm}}$, the typical field-driven evolution of the magnetic state is shown in Fig. 7, where $R = 150\,{\text{nm}}$. The *Q*-curve is different from the case in Fig. 6. In this region, there is no formation of skymion during the hysteresis process. Starting from the saturated ferromagnetic state (a), a stripe domain (b) is gradually formed in the curved film with the magnetic field increasing from − 1.5 to − 0.29 T. The topological number almost doesn’t change in this process. When the magnetic field is greater than − 0.29 T, the topological number decreases first and then increases to about 1 (c–f). Although the topological number is about 1, it doesn’t indicate that a skyrmion is formed, it is just originated from the two ends of the nanostripe. At *H*_*z*_ = 0.56 T, the sudden drop of *Q* from 1 to 0 indicates the disappearance of nanostripe and formation of the ferromagnetic state (g, h).

For $R < 150\,{\text{nm}}$, the typical field-driven evolution of the magnetic state is shown in Fig. 8, where $R = 60\,{\text{nm}}$. This dynamical process is similar to the case in nanotubes. At *H*_*z*_ = − 1.5 T, all magnetizations in the curved film uniformly point to the *z*-axis (a), and the topological number is zero. The ferromagnetic state stays until *H*_*z*_ increases to − 0.16 T. Then, stripes appear in the system, and the stripes gradually twist (b–d). In this process, the value of *Q* shows a great change. It first increases to about 2.8 at *H*_*z*_ = − 0.09 T, and then decreases to − 3.93 at *H*_*z*_ = 0.17 T. We can find four skyrmions are formed in the curved film at *H*_*z*_ = 0.17 T, which is formed by broken of the helical stripes on the left and right sides of the curved film. This dynamical process is similar to the case in nanotubes. These skyrmions can stay in a wide field range between *H*_*z*_ = 0.17 T and 1.29 T. At last, at *H*_*z*_ = 1.3 T, the skyrmions annihilate, the configuration of the curved film changes to the ferromagnetic state.

From the above description, we can find that the skyrmions are evolved from stripes. The curved radius has a big influence on this transition state. For large radius, the curvature has a little influence on the system energy, it shows maze domains. However, when the radius is less than 600 nm, the shape anisotropy induced by the curvature stretches the stripe to straight, and the stripes that parallel to the x-axis are preferred. These straight stripes cannot evolve to skyrmions. For small radius, the effect of the curvature becomes more obvious, the stripes twist along the surface of the curved film, the twisted stripes break to skyrmions. Thus, we can say the curvature influences the shape of the stripes that formed in the curved film, so three different kinds of evolutionary processes are found.

**Figure 9:**
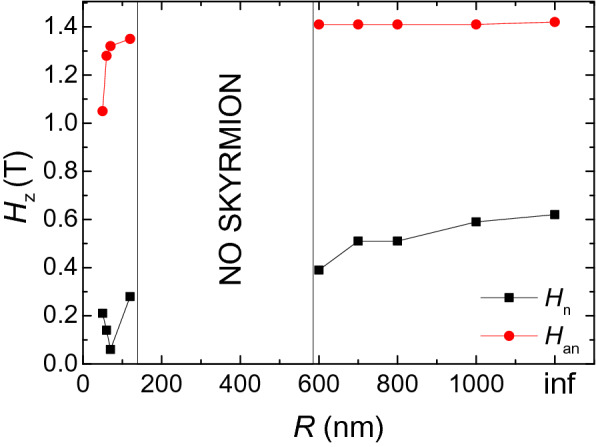
The nucleation field and annihilation field in curved films as a function of *R*.

The curvature also influences the nucleation and annihilation fields of skyrmions. Figure 9 shows the radius dependence of the nucleation and annihilation fields of skyrmions in the circular curved magnetic film. For $R > 600\,{\text{nm}}$, the nucleation field *H*_n_ slightly increases with *R*, however, the annihilation field *H*_*an*_ doesn’t change. For $R < 150\,{\text{nm}}$, the nucleation field *H*_n_ is smaller than the case of $R > 600\,{\text{nm}}$. It has the smallest value (0.1 T) when $R = 70\,{\text{nm}}$.

We know that the challenge for using magnetic skyrmions in applications is to find room temperature-stabled skyrmions without external force. Recently, several experiments have demonstrated that ultrathin ferromagnetic/heavy metal multilayers are good structures that can host skyrmions at zero magnetic field and room temperature^42–44^, and its size and density also can be controlled by the shape and size of the nanostructure^44^. Our above results show an evidence that curvature is also a method to reduce the magnetic field to stabilize skyrmions. Thus, we can predict that if we use the suitable parameters, we may observe room temperature-stabled skyrmions at zero magnetic field, and we can control its stability by curvature. This is the next aim of our future study.

**Figure 10:**
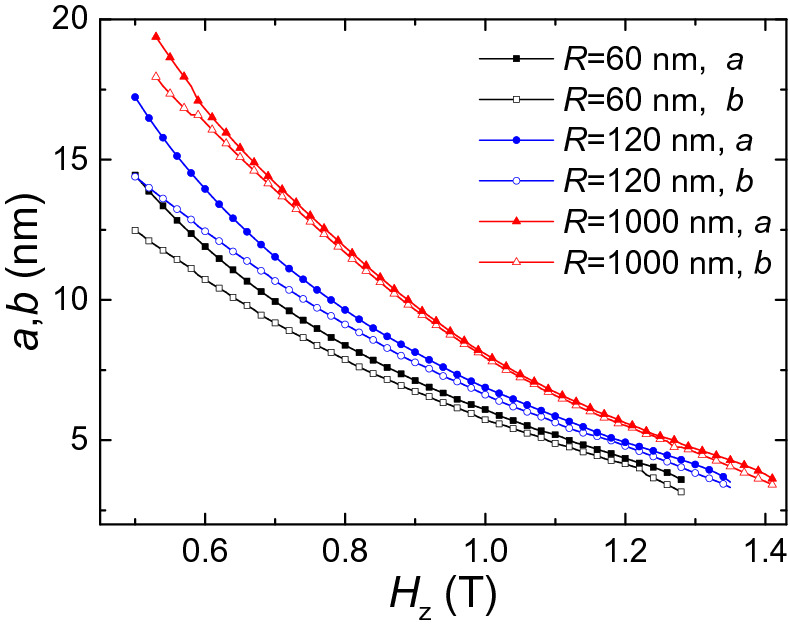
The size of skyrmion as a function of the magnetic field $H_{z}$ in curved films with different *R*.

The size of the skyrmion in the curved films also depends on *R*. Figure 10 shows the dependence of the skyrmion size on the applied magnetic field $H_{z}$ for different *R*. We also use *a* and *b* to denote the semimajor and semiminor axis of the ellipse. In curved film with larger *R* (*R* = 1000 nm), the value of *a* and *b* is almost equal. With decreasing of *R*, the difference between *a* and *b* becomes larger. Overall, the size of the skyrmion in curved film with smaller *R* is smaller than the ones in curved film with larger *R*.

## Conclusion

In summary, in this work we studied the magnetization evolution in three-dimensional chiral nanostructure, including nanotubes and circularly curved thin films. We found that the nucleation of skyrmion in nanotube is realized by broken of the helical stripes on the left and right sides of the nanotube without abrupt change of topological number. The nucleation field decreases, but the annihilation field increases with decreasing of the nanotube thickness. The shape of the skyrmion is a circular truncated cone. From the outer circular layer to the inner circular layer, the skyrmion becomes larger, and the deformation becomes more obvious. In curved magnetic films with fixed arc. There are three different kinds of hysteresis processes are found. For R > 600 nm, the behaviors are similar with the case of 2D thin film. For $150\,{\text{nm}} \le R < 600\,{\text{nm}}$, no skyrmion is formed in the hysteresis process. For R < 150 nm, the skyrmions are created by broken of the helical stripes on the left and right sides of the curved film.
